# Rethinking counterfeit medical supply chains: A critical review of the current literature

**DOI:** 10.1002/hcs2.97

**Published:** 2024-06-17

**Authors:** Iffath U. Syed, Travis W. Milburn

**Affiliations:** ^1^ Department of Health Policy and Administration The Pennsylvania State University Sharon Pennsylvania USA; ^2^ Department of Criminal Justice The Pennsylvania State University Sharon Pennsylvania USA

**Keywords:** criminal justice, drug prices, economic inequality, health inequity, health policy, medical supply chain, pharmaceutical industry, public health

## Abstract

The medical device and pharmaceutical industries include a range of drugs, machines, instruments, and apparatuses used to prevent, diagnose, treat disease and illness, or aid in rehabilitation for patients, and are expected to grow substantially in the coming years. However, they are often targets of criminal organizations who manufacture and profit from fraudulent products, infiltrating the market with counterfeit medical supply chains. In this paper, we discuss and analyze the extent and nature of this problem and make suggestions for mitigation and prevention of this worldwide challenge. Ultimately, we argue that a holistic approach is essential to addressing this problem, including the creation and dissemination of reliable and good quality data, developing healthcare systems to be more robust, establishing/enhancing intra‐ and international cooperation around this issue, and employing effective technological solutions, such as digital tracing.

## Graphical abstract

The medical device and pharmaceutical industries include a range of drugs, machines, instruments, and apparatuses used to prevent, diagnose, or treat disease and illness or aid in rehabilitation for patients, and are expected to grow substantially in the coming years. However, they are often targets of criminal organizations who manufacture and profit from fraudulent products, infiltrating the market with counterfeit medical supply chains (CMSCs). In this paper, we discuss and analyze the extent and nature of CMSCs, make suggestions for mitigation and prevention of this worldwide challenge, while demonstrating that a holistic approach is essential.

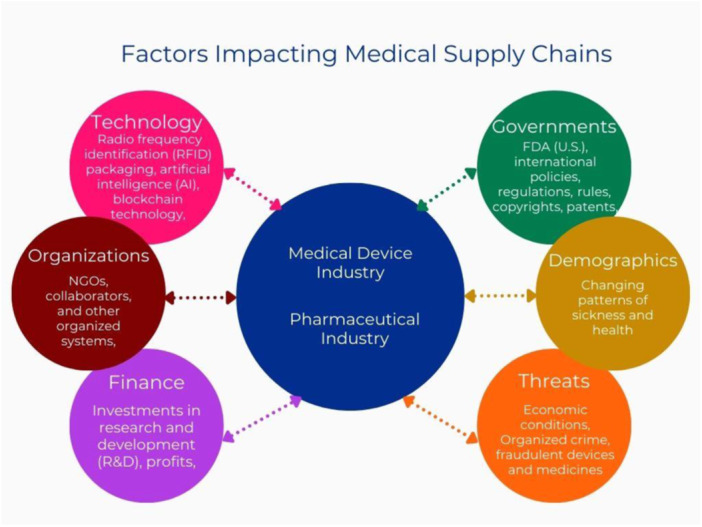

**Abbreviations**
CHIP: Children's Health Insurance Program
CMSCs: counterfeit medical supply chains
COVID‐19: novel coronavirus

## 1 INTRODUCTION

One of the largest issues facing the healthcare industry today is the global trade in counterfeit pharmaceutical products and medical devices in the United States and around the globe. For instance, 10% of medical products in middle‐ and low‐income nations are counterfeit or substandard [1] and is estimated to cost over the United States $30 billion annually [2]. Considering the expected 5% compounded growth of the medical device industry through 2028 [3], this problem may persist indefinitely. The purpose of this article is to address the significant issue of counterfeit pharmaceutical products and medical devices within the healthcare industry, both in the United States and globally.

The article delves into the various aspects of counterfeit pharmaceutical products and medical devices, including their prevalence, consequences, contributing factors, and responses from various stakeholders such as firms, governments, and intergovernmental organizations. It begins by providing a scope of the problem, with descriptions of definitions, characteristics, and segments of legitimate products. For instance, legitimate pharmaceutical industry includes generic, biopharmaceutical, and innovative segments, while medical devices consist of medical equipment and surgical appliances, with each segment serving distinct purposes within healthcare. Then, the focus of the article shifts to the global regions of origin regarding trafficking of counterfeit products, with major contributors from developing countries, describing consequences, challenges and continuing issues such as health dangers, reduced confidence in healthcare systems, and financial losses. Finally, it details response efforts, solutions, and future directions, including continued data collection, international cooperation, advocacy in reducing social inequalities and health inequities, and leveraging technological innovations.

Counterfeit pharmaceutical products and medical devices are those that are deliberately mislabeled as to their identity or source; and they include products which may contain no active ingredients, insufficient active ingredients, incorrect ingredients, or fake packaging [4]. Such products and devices, also known as falsified medical products, are defined by the World Health Organization as those “that deliberately/fraudulently misrepresent their identity, composition, or source” [1]. Falsified products may have any of a slew of deficient qualities, such as being produced in unsanitary environments or by personnel not qualified to make medical products, an incorrect or nonexistent active ingredient, toxicity, and/or the presence of chalk, potato starch, or corn starch. Since falsified products are often nearly indistinguishable in appearance from authentic ones [5], detection of counterfeits is often difficult [1].

Legitimate pharmaceutical products include three major segments—generic pharmaceuticals, biopharmaceuticals, and innovative pharmaceuticals [6], while medical devices include two major categories—medical equipment and apparatuses as well as surgical appliances and instruments [7]. Among pharmaceutical products, the generic segment includes copies of brand name pharmaceutical drugs with the same active ingredient, strength, dosage form, and route of administration [6], while the biopharmaceutical segment includes biological or cell‐based drugs. Finally, the innovative pharmaceutical segment refers to newly produced, chemically derived drugs that have been developed after extensive research and development (R&D) efforts and have undergone clinical trials in both humans and animals [6]. For medical devices, the medical equipment and apparatuses category consists of electromedical equipment and irradiation apparatuses, and includes a variety of powered devices, such as pacemakers, patient‐monitoring systems, magnetic resonance imaging machines, and ultrasonic scanning devices, while the surgical appliances and instruments category includes various appliances, apparatuses, and instruments used in anesthesia, surgery, orthopedics, and it includes artificial joints and limbs, hydrotherapy appliances, blood transfusion devices, and hypodermic needles [6].

## 2 WHERE DO COUNTERFEIT PHARMACEUTICAL PRODUCTS AND MEDICAL DEVICES COME FROM?

Counterfeit pharmaceutical products and medical devices are believed to be produced and manufactured from all around the world, particularly in Russia, Nigeria, the Philippines, Pakistan, Egypt, Indonesia, China, India, and Mexico [5, 8, 9]. Seventy‐five percent of global cases of counterfeit medicines originate from India where counterfeit drugs are estimated to represent 13%–30% of its pharmaceutical market—in fact, in India's major cities, one in every five medicines that sold is a fraudulent one [8]. India is also the major exporter of counterfeit drugs to less developed countries such as Nigeria, including antihuman immunodeficiency virus drugs [8]. In Nigeria, around 70% of drugs are imported, mostly from India, and it is alleged that some Nigerian drug importers have worked with some Indian manufacturers to produce fake and substandard drugs at a cheaper rate with less active ingredients which contributes to the problem of counterfeit medical supply chains (CMSCs) [9].

Transnational organized criminal groups are major contributors to the production of fraudulent medicines and devices, and are involved in all facets of the problem, especially trafficking. Indeed, counterfeiting is a lucrative enterprise for organized crime and provides an estimated $250 billion annually, a portion of which is counterfeit pharmaceuticals and medical devices [10]. These groups rely on the low costs of producing counterfeit goods and the high demand for such products. They leverage the same methods and routes used in other forms of trafficking to skirt detection, such as use of the dark web, and exploitation of legislative or justice system gaps [11]. Once these CMSCs enter the legitimate supply chain, they end up in direct street sales, or infiltrate well‐known wholesalers and retailers [5]. Recent examples of counterfeit devices include fraudulent thermometers, insulin pens, aortic pumps, hearing aids, pacemakers, respirators, and blood glucose meters [3]. In addition, there have been widespread cases of paracetamol cough syrups and teething products prepared with diethylene glycol (a toxic chemical used in antifreeze) that have been linked to 89 deaths in Haiti in 1995 and 30 infant deaths in India in 1998 [12, 13].

## 3 CONSEQUENCES OF CMSCS

Although the issue of CMSCs is multilayered, it has several consequences. The first set of consequences affects consumers and practitioners who are at risk across three key categories because (1) these products have no safety guarantee; (2) there is no proof of efficacy; and (3) purchasing such products is financially precarious [14]. More specifically, these risks and consequences include danger to human health and well‐being; failing to treat the conditions of patients; reduced confidence in health systems [1]; loss of income/profits to the rightful patent holder (pharmaceutical company/drug or medical device manufacturer); liability imposed on the healthcare industry (such as legal and cost‐control measures); and decreasing brand credibility [5]. These consequences have been steadily increasing since the 1990s, which means that firms who wish to recoup the costs of marketing their products and for R&D may have less incentives to innovate and produce new products given these CMSCs [5]. This latter consequence would be most impactful because the profitability driver in this industry is the ability to discover and market new drugs [6].

## 4 HOW ARE FIRMS, GOVERNMENTS, AND OTHERS RESPONDING TO CMSC?

Firms, governments, and other relevant entities are responding to CMSCs in a variety of ways. First, firms are using microtags such as radio frequency identification and enhanced packaging, such as the use of holograms to track the supply chain and distribution of their legitimate products and verify the shipment and sale of goods. Second, governments are responding to CMSCs with policies and regulations, including domestic and international policies that aim to prosecute criminal activity and enforce patent rights [5]. An effective regulatory structure is vital to guarantee competent supervision of medical devices to both prevent their production and catch them during their distribution. Such a system is responsible for reactions to the problem as it is recognized, but also ones that are preventative in nature [11]. Third, governments have passed legislation and implemented regulatory structures to deal with this issue. In the United States, the Food and Drug Administration is responsible for maintaining a safe drug and medical device market. The agency investigates relevant illegal activities, provides written warnings to violators, hosts summits, and educational forums and content for medical professionals and consumers, analyzes products, and works with other relevant agencies on prevention (such as US Customs and Border Protection) [15]. In Canada, the Health Products and Food Branch has a similar role in the identification, assessment, and management of counterfeit medical products with an emphasis on the removal of such products from the market [16]. At the state level, there are generally many entities with roles in the detection and mitigation of this problem and effective risk management should facilitate assistance from appropriate experts and agencies which include both industry and governmental actors [11].

Fourth, intergovernmental organizations and initiatives have attempted to address this challenge. The ongoing CMSC threat, which was especially pronounced during the COVID‐19 pandemic, serves as a useful example here. An Interpol‐led operation in March 2020 seized counterfeit medical products such as testing kits and face masks, worth over $14 million in 90 countries [17]. Given the risk of emerging communicable diseases and future pandemics, CMSCs will continue to be issues that will plague global markets. Crucially, this phenomenon is a transnational one that requires efficacious cooperation between states and their relevant entities for both prevention and adjudication of the problem, to include investigations (which may be joint given the nature of these supply chains), extradition, communication, and legal aid [11].

## 5 WHY DO CMSCS CONTINUE TO BE PROBLEMATIC DESPITE EFFORTS TO COUNTERACT THEIR PRESENCE?

CMSCs continue to be a problem for several reasons. First, the inherent nature of the healthcare and pharmaceutical industry, along with the high profit margin/high costs make it vulnerable to becoming a target for criminal activity. The primary demand driver in the industry is the desire to cure illness and disease, and some people are willing to pay premium prices to pursue this. Researchers also suggest that R&D costs of current medical and healthcare products are increasing while new product manufacturing is decreasing, meaning that firms are unable to generate effective products that can replace current products with expiring patents [18] due to attrition. Given this dilemma, firms respond by selling products at higher price points [19], which also creates a separate demand for inexpensive products [20] for consumers who are unable to afford the ever‐increasing costs of these products, or the costs of healthcare in general.

Consumer purchase decisions in the United States may be influenced, in part, by a lack of universal health insurance or other health inequities. For example, as of 2022, 7.3% of Americans or 26 million people did not have health insurance [21, 22]. While this is a record low, it is likely because of increased reliance and eligibility on public health insurance which occurred because many people became eligible for Medicaid and Children's Health Insurance Program (CHIP) due to lay‐offs and provisions in the *Families First Coronavirus Response Act* that required states to ensure continuous enrollment [21]. Recent research undertaken during the COVID‐19 pandemic suggests that while 57.8% of study participants from a sample in Miami, Florida had commercial insurance, others relied on government‐funded health insurance such as Medicare (27.2%), Medicaid (11.9%), and some individuals did not have any type of insurance coverage (1.6%) [23, 24]. While enrollment in Medicaid and CHIP grew by 29.8% during the COVID‐19 pandemic from February 2020 to December 2022; now that the COVID‐19 emergency ended in May 2023, people's eligibility and coverage for public health insurance, such as Medicaid, is expected to change and possibly worsen [21].

Another health equity issue that may influence drug purchases, including exposure to the CMSC market, is socioeconomic factors, such as income and education. In the United States, the federal minimum wage is set at $7.25 which results in earnings of about $15,000 a year for a full‐time worker [24]. Given that the share of waged labor's contribution to the US national income has been declining, while high‐income earners have been experiencing exponential increase in their salaries, America's income inequality gap continues to increase [25]. For example, between 1979 and 2007, the top 1% of income earners experienced a 200.5% increase in their income while the average income of the other 99% of US taxpayers grew by 18.9% [26]. The average annual income of an American grew only about 1.1% from 1979 to 2005 [27]. On the other hand, executive/CEO average compensation doubled from 1993 to 2003 [28]. High‐income earners, such as CEOs, had their compensation and stock growth increase 876% from 1978 to 2012, which is higher than the increases experienced by the S&P 500 (344%) and Dow Jones (389%) for the same period [27]; and US CEO compensation was 44 times that of the average worker [29]. While average weekly earnings of people in the United States varies considerably, it would still a source of health inequity between different socioeconomic groups. For example, weekly mean wages of men and women with high school education is $1098.95 and $833.83, respectively, whereas for those with a bachelor's degree, it is $2244.29 and $1508.86, respectively [30]. Increasing minimum wages has positive impacts on health insurance, as higher minimum wages are linked to a decrease in uninsured rates [23], which could dampen consumer exposure to CMSCs. Given these social and economic inequalities that drive health inequities, the parallel demand for cheap products is where CMSCs and organized crime may look to profiteer in the industry. It is further argued that technological innovation, and the quest for making huge profits in this industry make it unlikely that counterfeiting will end any time soon, or if it will ever be under control [5].

Another reason that CMSCs continue to be a problem is because the pharmaceutical and medical devices industries are highly concentrated. “The production in this industry is dominated by a small number of large firms that are able to shape the industry's direction and price levels” [6]. An additional attribute of this industry is that nearly all pharmaceutical products are produced and manufactured outside of the United States [31] (which means that the United States depends on foreign production of goods and a foreign/global supply chain. Mexico, for example, is a key country inadvertently involved in the counterfeit drug trade [32]. Criminal outlets in Mexico obtain their raw materials from China and India and then smuggle products into the United States through the southern border and sold as authentic medicines in pharmacies along the region [33]. In fact, there have been repeated Food and Drug Administration warnings about US consumers purchasing counterfeit drugs like Lipitor, Viagra, and Evista in border towns [5].

Furthermore, there are inherent changing demographics in the United States and around the globe, which includes aging populations, that may result in the exploitation of vulnerable groups by the CMSC market. The literature suggests that older adults often use multiple medications, called polypharmacy [34], which resulted in $234.1 billion in expenditures in 2008, and was more than double what was spent in 1999 [35], meaning that criminal structures operating CMSCs could also reap huge profits. Indeed, the cost of just one CMSC offense related to trafficking of misbranded and counterfeit drugs, specifically promethazine‐codeine cough syrup was approximately $53 million [36].

Finally, evolving medical and pharmaceutical technologies, global advancements in distribution and manufacturing, as well as ease of connectivity, and the presence of self‐diagnoses and self‐prescribing [37], make falsified medical products a significant concern. The United States is unique in its position in that it is the only country in the world where there is direct‐to‐consumer marketing of medical and pharmaceutical products [31]. While this empowers consumers, others think that it is also confusing for patients [38]. Consumers in the United States are also increasingly seeking the convenience of online shopping, including online pharmaceutical delivery, which is disruptive to traditional brick‐and‐mortar pharmacies and makes consumers vulnerable to counterfeit medicine [15] and organized crime, including identity theft. Indeed, counterfeit drugs are usually purchased and sold through traditional distribution channels, either to pharmacies, direct to consumers through mail‐order catalogs, or via the Internet [5].

## 6 SOLUTIONS AND FUTURE DIRECTIONS

To address the problem of CMSCs, we believe that a holistic approach is needed that considers the underlying root causes of these issues, which includes addressing socioeconomic inequalities and health inequities so that consumers who may be seeking cost‐effective alternatives for their healthcare are not inadvertently becoming victims of CMSCs. We also argue that while control measures at the Canadian‐United States and Mexican‐United States borders have been somewhat successful in thwarting CMSCs in North America, both US state and federal governments can also be involved in greater prevention measures as opposed to focusing predominately on reactionary efforts against CMSCs. For example, strengthening Medicaid and Medicare may seem, at first glance, as though they are disconnected to CMSCs, but these policies can result in safer supply chains and thwart profiteering from unauthorized channels. A strong support of governments for the development of the generic drug industry might also help to solve the problem. At the community level, a possible strategy that might be helpful in addressing the problem of CMSCs is collecting information and controlling access to drug purchases, for example, potentially vulnerable people might include children or grandchildren of elderly patients who purchase drugs for elderly that live alone. This is especially important since research shows that the primary sources of prescription drugs on the street included prescriptions to elderly patients [39]. Allowing community caregivers and management personnel to purchase drugs for these seniors might be an alternative strategy that could be beneficial and curb the abuse of prescription drugs intended for elderly patients.

To fully understand, and thus mitigate the problem of CMSCs, several other strategies are summarized here. First, efforts should be directed to collect and disseminate data on CMSCs, particularly concerning the circumstances surrounding events that involve counterfeit medical products and the root causes, prevalence, and effects of falsified medical products are necessary, and should be adequately supported [11]. This should include national and international cooperation—legal and law enforcement, restitution, whistleblower protections, and so forth [11]. There should also be increased screenings of drugs at the ports of entry, and the necessary training and resources to assist the Department of Homeland Security which is responsible in helping to manage threats and hazards that could impact the supply chain [40].

Second, efforts should also be directed to continue to advocate for universal healthcare and strengthen Medicaid and Medicare by reducing barriers in income eligibility thresholds so that at‐risk populations gain access so that vulnerable people can use legitimate sources of health and social care rather than relying on online pharmacies/the Internet or other avenues that may pose increased risks in accessing quality and legitimate care. This will also ensure that healthcare is managed by healthcare experts and does not leave healthcare in the hands of vulnerable consumers who are driven primarily by price incentives. Strengthening pharmaceutical policy is currently being carried out by the Biden administration through the *Inflation Reduction Act* of 2023 so that the government now has the authority to negotiate lower prescription pharmaceutical prices, which countries with universal healthcare systems have been doing for decades [41]. Indeed, it is suggested that distributors of medical supply chains who source drugs from countries such as Canada, which has universal healthcare and where prices are negotiated by the government, into free market systems such as the United States, enables profiteering from unauthorized channels [5]. Thus, having prenegotiated lower prices would thwart such unauthorized efforts, while also keeping transparency and checks and balances in a for‐profit industry that has seen previously unchecked exorbitant price fluctuations. As noted recently by the media, the prices of many types of medications have increased significantly more than inflation. For example, Fluconazole, which is an antifungal medication used to treat fungal infections, went up 1100%, while the price of Lisinopril, which is a drug to treat high blood pressure, went up 539% [42]. Previously, the price of Daraprim, which helps to fight parasitic infections, went up 5400% from $13.50 to $750 a tablet while the price of Isuprel [43], which is a medication used to treat heart conditions, was raised by 525%; and in similar circumstances, the price of Nitropress, which is a medication used to lower blood pressure, was raised 212% [44].

Third, given the complex global nature of medical supply chains, both legitimate and counterfeit, efforts to prevent and mitigate CMSCs will require national and international cooperation, as previously noted. This applies to regulatory and law enforcement efforts which must respond to the problem. The United Nations Office of Drugs and Crimes makes several suggestions in addition to regulatory and law enforcement recommendations [11]. Satisfactorily handling this growing problem should also include victim compensation, whereby states should install or improve upon processes to provide restitution and other forms of compensation to victims of these offences, and whistle‐blower safeguards (also known as protected disclosure) should be used to protect the identity and rights of those that make offences known to authorities. Combined with regulatory and law enforcement approaches, it is clear that new or refined legal codes will be needed to adequately confront this challenge [11]. Policy measures should also be generated in developed countries versus developing countries, because CMSCs tend to be worse in developing countries compared to developed countries due to poor regulatory oversights, low economic affordably, and poor drug quality standards. For example, in Nigeria, an ineffective judicial system and widespread corruption are major reasons why it is easy to produce and sell fake drugs, which has resulted in their proliferation and consumption resulting in treatment failures, worsening chronic conditions, organ dysfunction, damage, and the deaths [9]. In 1989, for instance, over 150 children died as a result of a formulation error in a drug [9]. In 2007, 82 truckloads of fake, banned, and expired drugs were seized by authorities and five fake drug warehouses were closed in Nigeria [9]. Storage and warehousing issues are also problematic when medicines are often left under the sun which could facilitate the deterioration of the active ingredients [9].

Finally, in the wake of increased concerns about technology and artificial intelligence, there are also some technological solutions to the problem of CMSCs. While technology has been vital in improving outcomes in healthcare and producing a more robust supply chain of medical devices and products, simultaneously, technology has similarly made counterfeiting and circulating fraudulent devices and products easier as well [45]. One possible solution to combatting CMSCs is to rely upon novel or updated technologies to improve supply chains as they become available. In this vein, a potential technological solution is to deploy digital tracing throughout medical supply chains. One specific digital tracing suggestion is to use blockchain technology through a nonfungible token to create a decentralized storage system for consistent, dependent, and efficient medical devices traceability capacity and ownership management system [45]. Such a system would digitally represent individual medical devices, record their attributes, and keep track of their metadata during all stages of production, distribution, and consumption. A similar technological solution offered by U‐NICA involves the use of security labels which allow for digital tracing, monitoring, and recording which is accessible through an app [3].

## 7 CONCLUDING REMARKS

In this paper, we have highlighted the various problems stemming from counterfeit pharmaceutical products and medical devices, as well as policies and interventions aimed to respond to these issues. While some of the interventions and policies we have mentioned, namely regulatory practices and inspections, have been successful, we note some policy challenges that continue to be problematic, with an overall need to place resources and efforts towards prevention measures directed towards root causes. We have advocated for the creation and dissemination of high‐quality research and data, the (re)development of healthcare systems to be more robust and equity‐oriented, establishing/enhancing intra‐ and international cooperation, and employing effective technological solutions. Our novel analysis is an interdisciplinary approach from the fields of public health, as well as criminal justice and security studies that seeks high quality, regulatory, health, and safety compliance in supply chain management, but also one that addresses global challenges and issues such as economic vulnerability at the level of individuals and communities. This is especially important in the wake of pandemic‐level events, and places the onus of responsibility on structures that should minimize socioeconomic inequality and health inequities, for example by making drugs more affordable for individuals, reducing barriers in income eligibility thresholds for publicly funded insurance, such as Medicare and Medicaid, so that more vulnerable people gain access, and by addressing social determinants of health, such as increasing wages to dampen the effect of income inequality, and compressing the gaps between low and high‐income earners.

## AUTHOR CONTRIBUTIONS

**Iffath U. Syed**: Conceptualization (lead); visualization (equal); writing—original draft (equal), writing—review and editing (equal); project administration (lead); **Travis W. Milburn**: Writing—original draft (equal); writing—review and editing (equal).

## CONFLICT OF INTEREST STATEMENT

The authors declare no conflict of interest.

## ETHICS STATEMENT

Not applicable.

## INFORMED CONSENT

Not applicable.

## Data Availability

Data sharing are not applicable to this article as no data sets were generated or analyzed during the current study.
